# Influence of Duffy antigen receptor for chemokines on HIV infection

**DOI:** 10.1186/1742-4690-10-S1-O31

**Published:** 2013-09-19

**Authors:** Robin Weiss

**Affiliations:** 1 Division of Infection & immunity, University College London, London, UK

## Background

Duffy antigen receptor for chemokines (DARC) binds inflammatory chemokines including several that also bind to CCR5. A recessive DARC allele which blocks expression on red cells affects ~90% of sub-Saharan Africans but very few people of non-African origin. Approximately 50% of DARC null people show African benign neutropenia. We have investigated whether DARC acts as an HIV co-receptor and whether the null phenotype affects risk of HIV infection.

## Materials and methods

DARC was expressed in CD4+ cells to test if it acts as an attachment or entry co-receptor for HIV-1 and HIV-2. Genotyping of cohorts exposed to HIV-1 with respect to DARC FyA, FyB and Fy null alleles was conducted to assess its affect on neutropenia, CCL5 plasma levels and relative risk of HIV-1 infection.

## Results

HIV-2 strains but not HIV-1 strains utilised DARC as an entry co-receptor. However, HIV-1 could bind to DARC on red cells and be delivered to activated CD4+ T-cells resulting in subsequent infection. GWAS analysis confirmed the link between DARC null genotype and African benign neutropenia. The DARC null phenotype with African benign neutropenia resulted in low CCL5 plasma levels and increased the relative risk of HIV-1 infection approximately 3-fold.

## Conclusion

The African-specific DARC null allele increases risk of HIV infection.

